# Circulating Exosomal miRNAs as Novel Biomarkers Perform Superior Diagnostic Efficiency Compared With Plasma miRNAs for Large-Artery Atherosclerosis Stroke

**DOI:** 10.3389/fphar.2021.791644

**Published:** 2021-11-26

**Authors:** Mengying Niu, Hong Li, Xu Li, Xiaoqian Yan, Aijun Ma, Xudong Pan, Xiaoyan Zhu

**Affiliations:** ^1^ Department of Neurology, The Affiliated Hospital of Qingdao University, Qingdao, China; ^2^ Institute of Cerebrovascular Diseases, The Affiliated Hospital of Qingdao University, Qingdao, China; ^3^ Department of Critical Care Medicine, The Affiliated Hospital of Qingdao University, Qingdao, China

**Keywords:** ischemic stroke, biomarkers, exosomes, miRNA, atherosclerosis

## Abstract

Recently, exosomal miRNAs have been reported to be associated with some diseases, and these miRNAs can be used for diagnosis and treatment. However, diagnostic biomarkers of exosomal miRNAs for ischemic stroke have rarely been studied. In the present study, we aimed to identify exosomal miRNAs that are associated with large-artery atherosclerosis (LAA) stroke, the most common subtype of ischemic stroke; to further verify their diagnostic efficiency; and to obtain promising biomarkers. High-throughput sequencing was performed on samples from 10 subjects. Quantitative real-time polymerase chain reaction (qRT-PCR) was performed on exosomes and plasma in the discovery phase (66 subjects in total) and the validation phase (520 subjects in total). We identified 5 candidate differentially expressed miRNAs (miR-369-3p, miR-493-3p, miR-379-5p, miR-1296-5p, and miR-1277-5p) in the discovery phase according to their biological functions, 4 of which (miR-369-3p, miR-493-3p, miR-379-5p, and miR-1296-5p) were confirmed in the validation phase. These four exosomal miRNAs could be used to distinguish LAA samples from small artery occlusion (SAO) samples, LAA samples from atherosclerosis (AS) samples, and LAA samples from control samples and were superior to plasma miRNAs. In addition, composite biomarkers achieved higher area under the curve (AUC) values than single biomarkers. According to our analysis, the expression levels of exosomal miR-493-3p and miR-1296-5p were negatively correlated with the National Institutes of Health Stroke Scale (NIHSS) score. The four identified exosomal miRNAs are promising biomarkers for the diagnosis of LAA stroke, and their diagnostic efficiency is superior to that of their counterparts in plasma.

## Introduction

Stroke is the leading cause of long-term disability and one of the leading causes of death worldwide and is a growing global burden (Barrington et al., 2017; Ornello et al., 2018; Raju et al., 2020; Zuo et al., 2020). Ischemic stroke accounts for 80% of stroke incidence (Tiedt et al., 2017). According to the Trial of Org 10,172 in Acute Stroke Treatment (TOAST), large-artery atherosclerosis (LAA) stroke is the most common type (Ornello et al., 2018). The primary cause of this type of ischemic stroke is narrowing or obstruction of the main brain trunk or cortical artery branches due to atherosclerosis (AS) (Adams et al., 1993). AS is a condition in which fatty and/or fibrous material accumulates in the lining of the arteries (Lu and Rothenberg, 2018). Proper diagnosis of ischemic stroke is important for subsequent treatment (Prabhakaran et al., 2015; Powers, 2020).

Previous studies have found that miRNAs play important roles in a variety of diseases (Rupaimoole and Slack, 2017). The expression of miRNAs varies greatly between normal and pathological states and can be disease-specific (Liu et al., 2019). miRNAs are endogenous molecules with lengths of 20–25 nucleotides that regulate gene expression after transcription (Lu and Rothenberg, 2018; Treiber et al., 2019). miRNAs have a wide range of functions in cells and can be released into the peripheral circulation in small extracellular vesicles or bound to proteins (Tiedt et al., 2017). miRNAs can serve as valuable diagnostic biomarkers in a variety of diseases, but studies have rarely been conducted on their use as biomarkers in ischemic stroke (Tiedt et al., 2017). In addition, miRNAs are unstable in the presence of RNase and are therefore easily degraded in plasma (Matsuura et al., 2016; Min et al., 2019). However, exosomes provide a relatively stable environment for miRNAs and can protect miRNAs from degradation, which has attracted considerable research interest (van Niel et al., 2018; Kalluri and LeBleu, 2020).

Exosomes are endosomal-derived phospholipid bilayer vesicles that are 40–160 nm (on average 100 nm) in diameter. Exosomes play very important roles not only in physiological processes, such as cell-to-cell communication, material transport, and the immune response, but also in pathological processes, such as cardiovascular and cerebrovascular diseases (Tiedt et al., 2017; van Niel et al., 2018; Kalluri and LeBleu, 2020). Exosomes contain a variety of substances, including noncoding RNAs such as miRNAs, mRNAs, and proteins (van Niel et al., 2018; Kalluri and LeBleu, 2020). However, scarcely any research has been performed on diagnostic biomarkers for ischemic stroke in exosomes (Kalluri and LeBleu, 2020).

The purposes of our study were to verify the association of exosomal miRNAs with LAA stroke, the major subtype of ischemic stroke, and to find promising diagnostic biomarkers. In this study, we characterized total miRNA profiles in plasma exosomes of LAA stroke patients and healthy controls using high-throughput sequencing. Furthermore, we identified exosomal miRNAs that distinguish LAA stroke patients from controls, validated these biomarkers in a large independent cohort, and compared their diagnostic performance with plasma miRNAs.

## Materials and Methods

### Participant Information and Sample Collection

A total of 596 subjects recruited from the Affiliated Hospital of Qingdao University Neurology Department from June 2018 to March 2020 were included in our study, including 10 for high-throughput sequencing, 66 for the discovery phase, and 520 for the validation phase. Patients were enrolled in our study cohort within 72 h of symptom onset when computed tomography (CT) or magnetic resonance imaging (MRI) demonstrated a new infarction. Patient diagnosis was based on the TOAST criteria and a combination of CT, MRI, and magnetic resonance angiography (MRA)/CT angiography (CTA) findings. The inclusion and exclusion criteria are shown in the Supplementary Materials (Glisic et al., 2018; Fernandez et al., 2019; Shen et al., 2019; Zuo et al., 2020). Informed consent was obtained from all participants. This study was approved by the ethics committee of Qingdao University Affiliated Hospital. All procedures followed were in accordance with the ethical standards of the responsible committee on human experimentation (institutional and national) and with the Helsinki Declaration as revised in 2013. Peripheral blood samples from each participant were collected in EDTA tubes following a regular venipuncture procedure in the morning under fasted conditions without water intake within 24 h of hospital admission. After centrifugation at 3,000 ×*g* for 15 min at 4°C, the plasma was immediately stored at −80°C until use.

### Exosome Isolation

For exosome isolation, we used total exosome separation reagent for plasma (Cat 4484450, Invitrogen Carlsbad, United States) (Tian et al., 2020). In brief, each plasma sample was centrifuged at room temperature for 20 min at 2000 × *g* to remove cells and debris and then centrifuged at 10,000 ×*g* for 20 min to remove debris for a second time. Then, 1 ml of plasma was added to 0.5 ml of phosphate-buffered saline (PBS). The sample was vortexed for thorough mixing; then, 50 μl of proteinase K was added to the mixture, and the mixture was incubated at 37°C for 10 min. Next, 300 μl of exosome precipitation reagent was added to the supernatant. After thorough mixing, the mixture was incubated for 30 min at 4°C and then centrifuged at 10,000 ×*g* for 5 min. The isolated exosomes were contained in the pellet at the bottom of the tube. Finally, the pellet was resuspended in PBS.

### Transmission Electron Microscopy

Exosome-enriched solution was placed on a copper grid and incubated at room temperature for 2 min. Then, the exosomes were placed in 2% phosphotungstic acid for 2 min and washed with sterile distilled water. The morphology of the exosomes was observed by using TEM (Hitachi, Tokyo, Japan) (Min et al., 2019).

### Nanoparticle Tracking Analysis

Exosome pellets were resuspended in 1 ml of PBS and examined with a ZetaView PMX 110 instrument (Particle Metrix, Meerbusch, Germany). We used NTA software (ZetaView) to analyze the particle size and quantity (Min et al., 2019).

### Western Blot Analysis

We extracted total protein from exosomes with RIPA and PMSF buffers at a ratio of 99:1 (MCE, United States), and the concentration of the total protein was normalized after a BCA assay was performed. The protein samples were then subjected to 10% SDS-PAGE and transferred onto a membrane. The PVDF membrane was incubated with primary antibodies against CD9, CD63, TSG101 and GRP94 (ab92726, ab134045, ab125011, and ab238126, respectively; Abcam, Cambridge, United Kingdom) at 4°C overnight and then with HRP-conjugated secondary antibodies (Abcam, Cambridge, United Kingdom) for an hour.

### RNA Isolation and RNA Analysis

According to a published protocol, we used an miRNeasy Serum/Plasma Advanced kit to extract and purify total RNA from plasma exosome-enriched fractions (Androvic et al., 2019; Peng et al., 2020). RNA concentration, purity, and integrity were assessed using the RNA Nano 6000 Assay Kit of an Agilent Bioanalyzer 2,100 System (Agilent Technologies, CA, United States) (Min et al., 2019).

### Library Preparation and Sequencing

A total of 5 µg of RNA was used as input material for RNA library preparation. Sequencing libraries were created with an NEBNext^®^ Ultra™ Directional RNA Library Prep Kit for Illumina R (NEB, United States) according to the manufacturer’s recommendations. First-strand cDNA was synthesized using random hexamer primers and M-MuLV Reverse transcriptase (RNase H). Then, second-strand cDNA was synthesized by using DNA polymerase I and RNase H. Once the 3′ ends of the DNA fragments were adenylated, they were attached to NEBNext adaptors with hairpin loop structures to prepare for hybridization. The library fragments were purified using an AMPure XP system (Beckman Coulter, Beverly, United States), and cDNA fragments with a length of 150–200 bp were selected. PCR was then performed using Phusion High-Fidelity DNA polymerase, universal PCR primers, and Index (X) primers. Finally, the products were purified (AMPure XP system), and the library quality was evaluated by using the Agilent Bioanalyzer 2,100 system.

The index-encoded samples were clustered using a cBot Cluster Generation System with a TruSeq PE Cluster Kit V3-cBot-HS (Illumina) according to the manufacturer’s instructions. After cluster generation, the Illumina HiSeq 4,000 platform was used for sequencing, and 150 bp paired-end reads were generated.

### miRNA Quantification and Differential Expression Analysis

First, the transcripts per million (TPM) values were used to normalize the raw counts. Transcripts with a *padj* (*p-*value after adjust) < 0.05 and a fold change (FC) > 2.0 or < −2.0 were considered to be differentially expressed. The differentially expressed miRNAs were visualized with volcano plots.

### Target Gene Prediction and Gene Ontology/Kyoto Encyclopedia of Genes and Genomes Pathway Enrichment Analysis

For all miRNAs that were differentially expressed between LAA stroke samples and control samples, the potential target genes that were predicted by both miRanda and RNAhybrid were included in subsequent analyses. We used DAVID and KOBAS to enrich the biological functions of the target genes (Low et al., 2019; Min et al., 2019).

### Quantification of miRNA Expression With qRT-PCR

Total RNA was extracted and purified from plasma exosomes according to the manufacturer’s protocols (Androvic et al., 2019). Reverse transcription of miRNAs was performed using a Mir-X miRNA First-Strand Synthesis Kit (Takara, Japan). TB-Green Premix Ex Taq™ II (Takara, Japan) was used for quantitative amplification. The expression levels of U6 were used to normalize the relative expression levels of miRNA, and the 2^-∆∆Ct^ method was used for quantification (Raoof et al., 2018). The primer sequences are listed in the Supplementary Materials.

### Statistical Analysis

Statistical analyses were performed using SPSS 22.0. The false discovery rate (FDR) was controlled for multiple comparisons, and *p* < 0.05 was considered to indicate a significant difference. Normally distributed data was presented as the mean ± standard deviation (SD) while non-normally distributed data was presented as the median (interquartile range). Statistical significance (*p* < 0.05) was determined by means of independent-samples t-tests, chi-square tests and Kruskal-Wallis tests. We constructed the regression model by binary logistic regression. Receiver operating characteristic (ROC) curves were generated, and the area under the curve (AUC) was also calculated to assess the diagnostic efficiency of candidate miRNAs.

## Results

### Participant Characteristics

In this study, a total of 596 subjects were recruited. Of these participants, 348 patients were diagnosed with ischemic stroke at a tertiary hospital, among which 193 patients were diagnosed with LAA and 155 patients were diagnosed with small artery occlusion (SAO). Another 105 patients were diagnosed with AS but did not develop stroke, and 143 of the subjects were healthy controls. The demographics and clinical features of the subjects are shown in Table 1. There were no significant differences in the age or sex distributions of the participants, which was consistent with the clinical manifestations (*p* > 0.05).

**TABLE 1:** Demographic and clinical characteristics of the subjects. **(**TG, triglycerides; TC, total cholesterol; LDL, low-density lipoprotein; HDL, low-density lipoprotein).

Characteristic	LAA (n = 193)	SAO (n = 155)	As (n = 105)	Controls (n = 143)	*p*-val
Age, mean (SD), y	62.9 (10.5)	62.9 (11.8)	63.4 (9.6)	64.7 (8.9)	0.38
Female, n (%)	88 (45.6%)	69 (44.5%)	55 (52.4%)	65 (45.5%)	0.61
Risk factors, n (%)					
Hypertension	122 (63.2%)	91 (58.7%)	57 (54.3%)	99 (69.2%)	0.08
Smoking history	58 (30.1%)	52 (33.5%)	28 (26.7%)	35 (24.5%)	0.34
Drinking history	56 (29.0%)	36 (23.2%)	23 (21.9%)	31 (21.7%)	0.37
Diabetes mellitus	57 (29.5%)	38 (24.5%)	26 (24.8%)	29 (20.3%)	0.27
TG, mean (SD)	1.4 (1.2)	1.3 (0.8)	1.5 (1.1)	1.4 (0.7)	0.06
TC, mean (SD)	4.3 (1.2)	4.2 (1.1)	4.8 (1.1)	4.7 (1.1)	<0.01
LDL, mean (SD)	2.4 (0.8)	2.6 (0.9)	2.5 (0.3)	2.9 (0.9)	<0.01
HDL, mean (SD)	1.2 (0.3)	1.3 (0.3)	2.6 (0.9)	1.3 (0.3)	<0.01
Hypertension med use (%)	18 (9.3%)	17 (11.0%)	35 (33.3%)	29 (20.3%)	<0.01
Diabetes med use (%)	11 (5.7%)	15 (9.7%)	21 (20.0%)	8 (5.6%)	<0.01
Lipid-lowing drug (%)	1 (0.5%)	3 (1.9%)	6 (5.7%)	4 (2.8%)	0.04
Anti-platelet drugs (%)	6 (3.1%)	1 (0.6%)	6 (5.7%)	10 (7.0%)	0.02

### Characteristics of Exosomes

**FIGURE 1:**
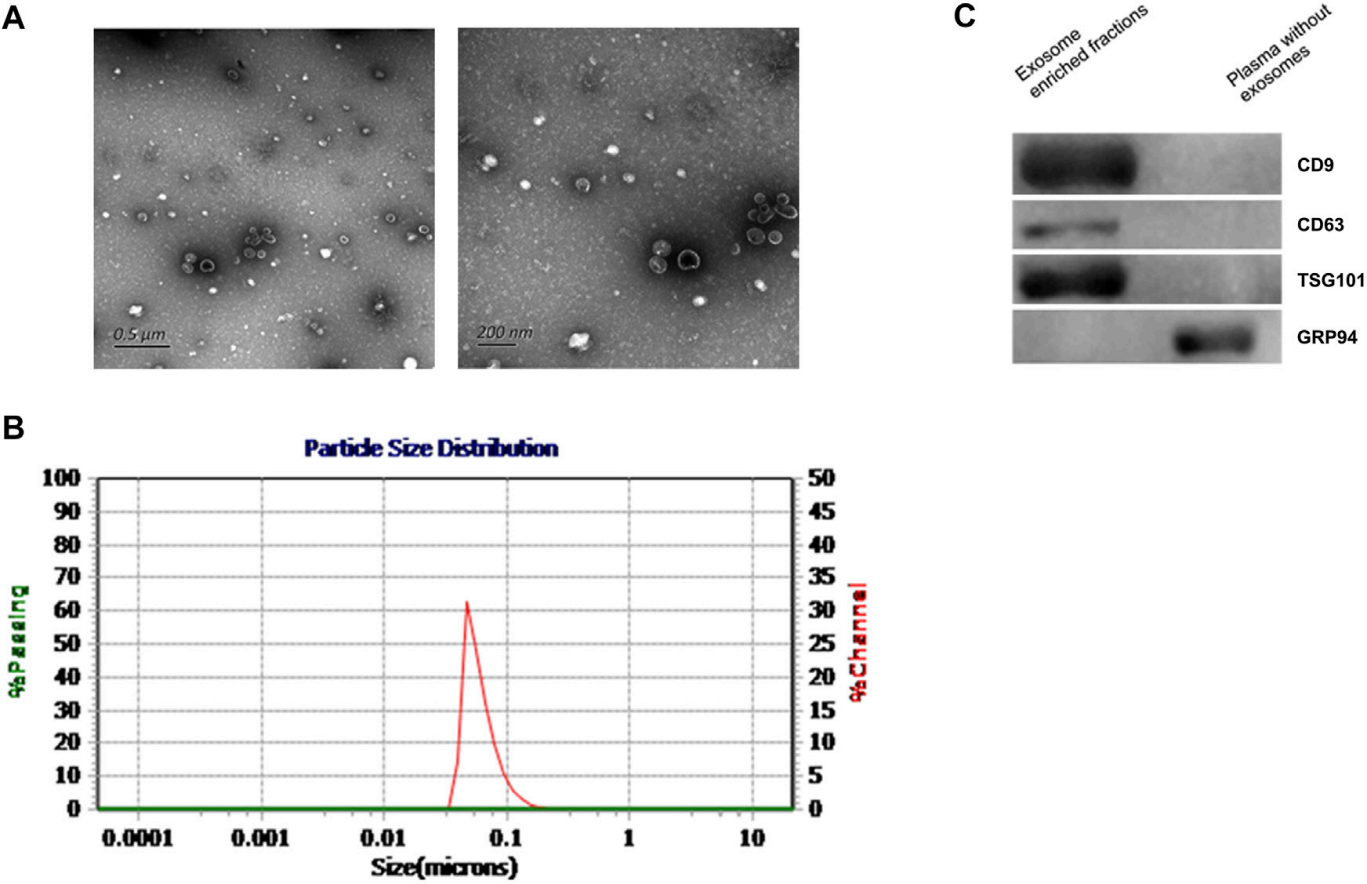
Characteristics of plasma exosomes from participants. **(A)**. TEM images showing that the exosomes were bilayer vesicles (left image: wide field containing multiple exosomes; right: close-up image of a single exosome). **(B)**. NTA results showing that the diameter of the enriched plasma exosomes was approximately 30–150 nm. **(C)**. Western blot showing that CD9, CD63 and TSG101 were detected in the enriched exosome samples isolated from plasma, while the negative exosomal marker GRP94 was not detected in these samples.

According to previous literature, we verified the enriched exosomes via TEM, NTA, and western blot analysis. We extracted and identified total plasma-derived exosomes according to the methods recommended by the International Society for Extracellular Vesicles (Théry et al., 2018). In this study, the exosomes we extracted were observed via electron microscopy to be phospholipid bilayer vesicles with an average diameter of 100 nm, and NTA showed that exosomes in diameter were 30–150 nm (Figures 1A,B). Western blotting verified 3 positive markers of exosomes (CD9, CD63, and TSG101) (van Niel et al., 2018; Kalluri and LeBleu, 2020). In addition, a negative marker of exosomes, GRP94, was absent (Figure 1C). The above findings suggested that we successfully obtained exosomes.

### RNA Sequencing Analysis

**FIGURE 2:**
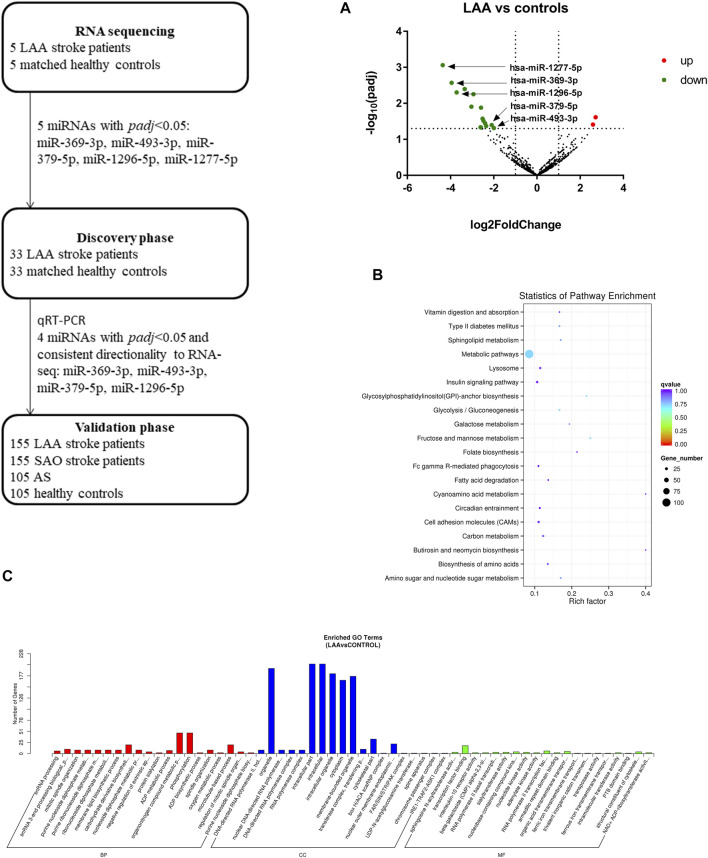
Workflow and RNA sequencing results. Target analysis of miRNAs differentially expressed in enriched exosome fractions from LAA and control plasma samples. GO/KEGG enrichment was performed on differentially expressed miRNAs identified in exosome-enriched plasma, and miRNA analysis was performed. **(A)**. Volcano map showing the high-throughput sequencing results for LAA vs. healthy control samples; red indicates upregulation, and green indicates downregulation. **(B)**. Bubble plot of the KEGG pathway enrichment results. **(C)**. Bar plot of the GO enrichment results (biological process, cellular component, and molecular function categories).

To obtain overall profiles of the miRNAs derived from exosomes in plasma, we performed high-throughput sequencing on 10 samples (5 LAA samples and 5 control samples) and obtained 830 miRNAs. Among the miRNAs there were 18 differentially expressed miRNAs (16 miRNAs were downregulated, and 2 miRNAs were upregulated) (Figure 2A). The functions of the genes targeted by these miRNAs were analyzed with the KEGG database, and the top 20 functions were enriched (Figure 2B). As shown in the figure, the function terms associated with the genes targeted by these miRNAs included the metabolic pathways, cell adhesion molecules (CAMs), and insulin signaling pathway terms, which suggests that the genes may be associated with the occurrence of disease. Through GO analysis, we found enrichment of numerous terms in the biological process category, such as the organonitrogen compound metabolic process and phosphorylation terms. The enriched terms in the cellular component category included the intracellular part, cytoplasm, membrane-bounded organelle and intracellular organelle terms. In the molecular function category, the enriched terms included the transcription factor binding and RNA polymerase II transcription factor binding terms (Figure 2C).

### Expression of Candidate miRNAs in Exosomes in the Discovery Phase

**FIGURE 3:**
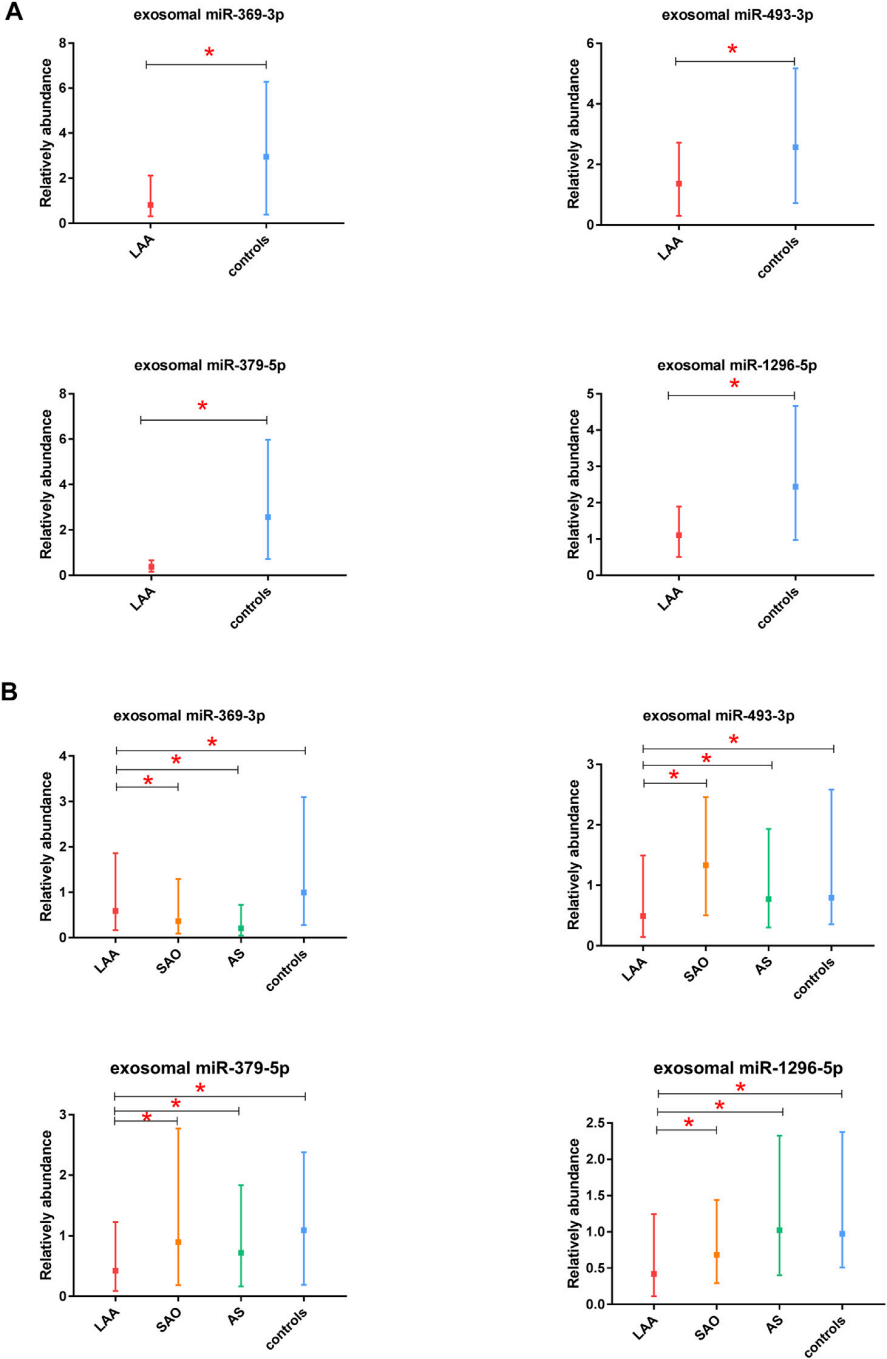
Discovery and validation of exosomal miR-369-3p, miR-493-3p, miR-379-5p, miR-1296-5p. **(A)**. Results from the discovery phase. **(B)**. Results from the validation phase (median ± interquartile range, Mann-Whitney *U* test. *p < 0.05).

In this study, we selected 5 miRNAs according to their biological functions and differential expression in the discovery phase: miR-369-3p, miR-493-3p, miR-379-5p, miR-1296-5p, and miR-1277-5p (|log2(FC)|>1, *p* < 0.05). These five miRNAs were all downregulated on the basis of previous sequencing results. A total of 66 subjects were recruited in the discovery phase (33 LAA subjects, 33 controls). Interestingly, we found significant differences in expression between the two groups for all miRNAs except miR-1277-5p (Figure 3A, Supplementary Figure S1). The other four miRNAs derived from exosomes were able to distinguish LAA subjects from control subjects (unadjusted raw *p*-value < 0.05) (Figure 3A). The sequencing results were verified with experiments in the discovery phase.

### Identification of Exosomal miRNAs as Candidate Biomarkers in the Validation Phase

In the validation phase, we verified the four exosomal miRNAs screened out as potential biomarkers in the discovery phase. miR-1277-5p was excluded because it did not perform well in differentiating the LAA group from the control group in the discovery phase. miR-369-3p, miR-493-3p, miR-379-5p, miR-1296-5p were ultimately selected for verification in 520 participants (310 ischemic stroke patients, 105 AS patients, and 105 healthy controls), and their expression levels were analyzed by qRT-PCR. To obtain the most robust and reliable biomarker, we further subdivided the stroke group into two groups: the LAA group (155 patients) and the SAO group (155 patients). In addition, we performed ROC curve analysis and calculated the AUC values to further confirm the diagnostic efficiency of these biomarkers. The results obtained were consistent with those from previous experiments: all four of the miRNAs could distinguish the LAA subjects from control subjects. Moreover, significant differences in the expression of exosome-derived miR-369-3p were observed in the LAA group vs. the SAO group and in the LAA group vs. the AS group. Likewise, exosomal miR-493-3p, miR-379-5p, miR-1296-5p showed the same differential expression patterns (*p* < 0.05) (Figure 3B). Intergroup analysis of exosomal miRNAs in LAA and SAO groups ruled out such changes due to acute ischemic stroke. While analysis of LAA and AS groups demonstrated that the differential expression of exosomal miRNAs was probably due to the rupture of atherosclerotic plaques.

**FIGURE 4:**
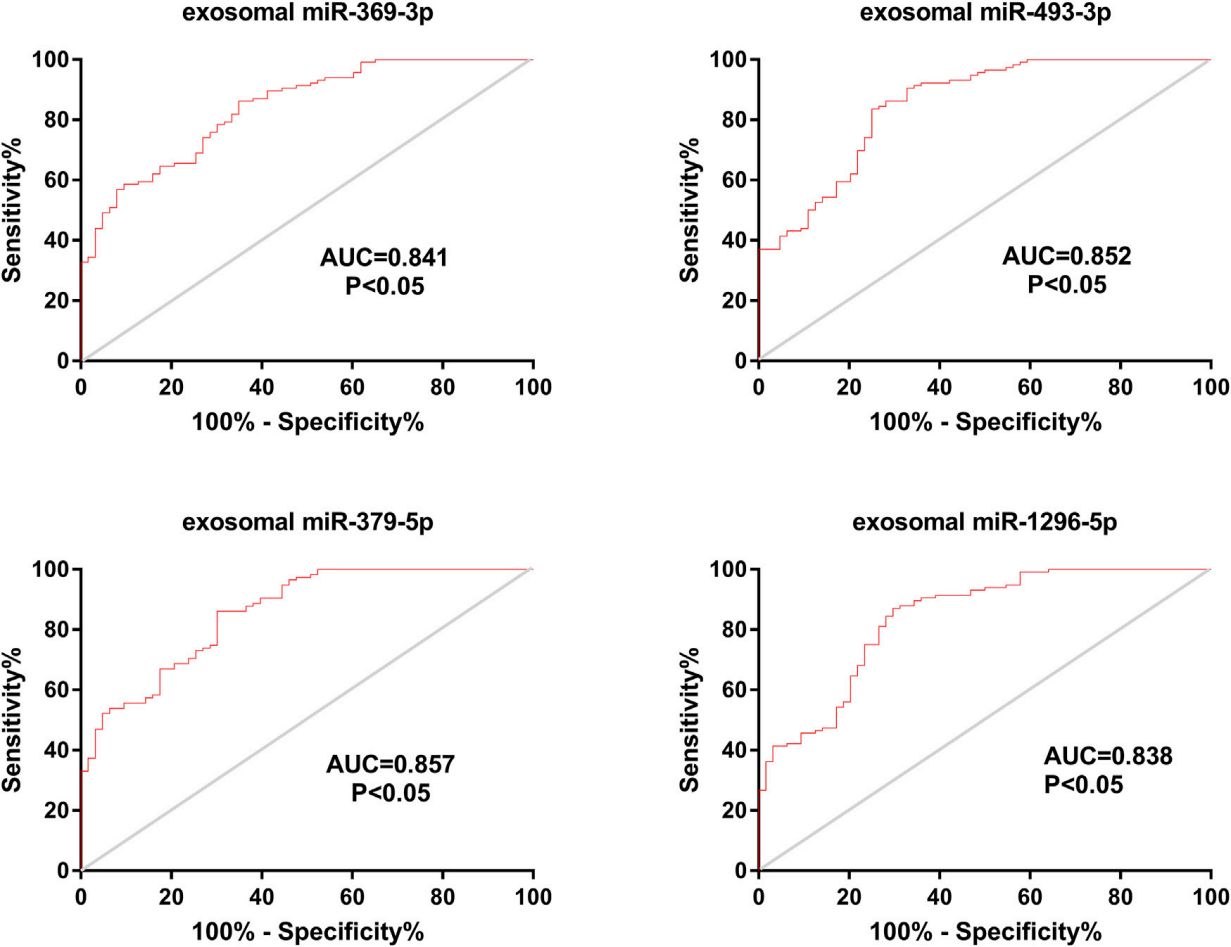
Diagnostic efficiency of exosomal miRNAs in the validation phase. ROC curve analyses were performed. The AUCs of the 4 exosomal miRNAs were calculated and are shown in red.

In addition, we calculated the AUC values for the four miRNAs. The AUC values for exosomal miR-369-3p, miR-493-3p, miR-379-5p and miR-1296-5p were 0.841, 0.852, 0.857, and 0.838, respectively (95% CI, 0.783–0.898; 0.793–0.910; 0.801–0.913; 0.777–0.900; *p* < 0.05) (78.46% PPV and 76.18% NPV; 83.15% PPV and 75.62% NPV; 80.82% PPV and 77.28% NPV; 81.23% PPV and 78.65% NPV) (Figure 4), which shows the potential value of these four miRNAs as biomarkers for discriminating patients with LAA from other groups.

### Comparison of Exosomal miRNAs and Plasma miRNAs

**FIGURE 5:**
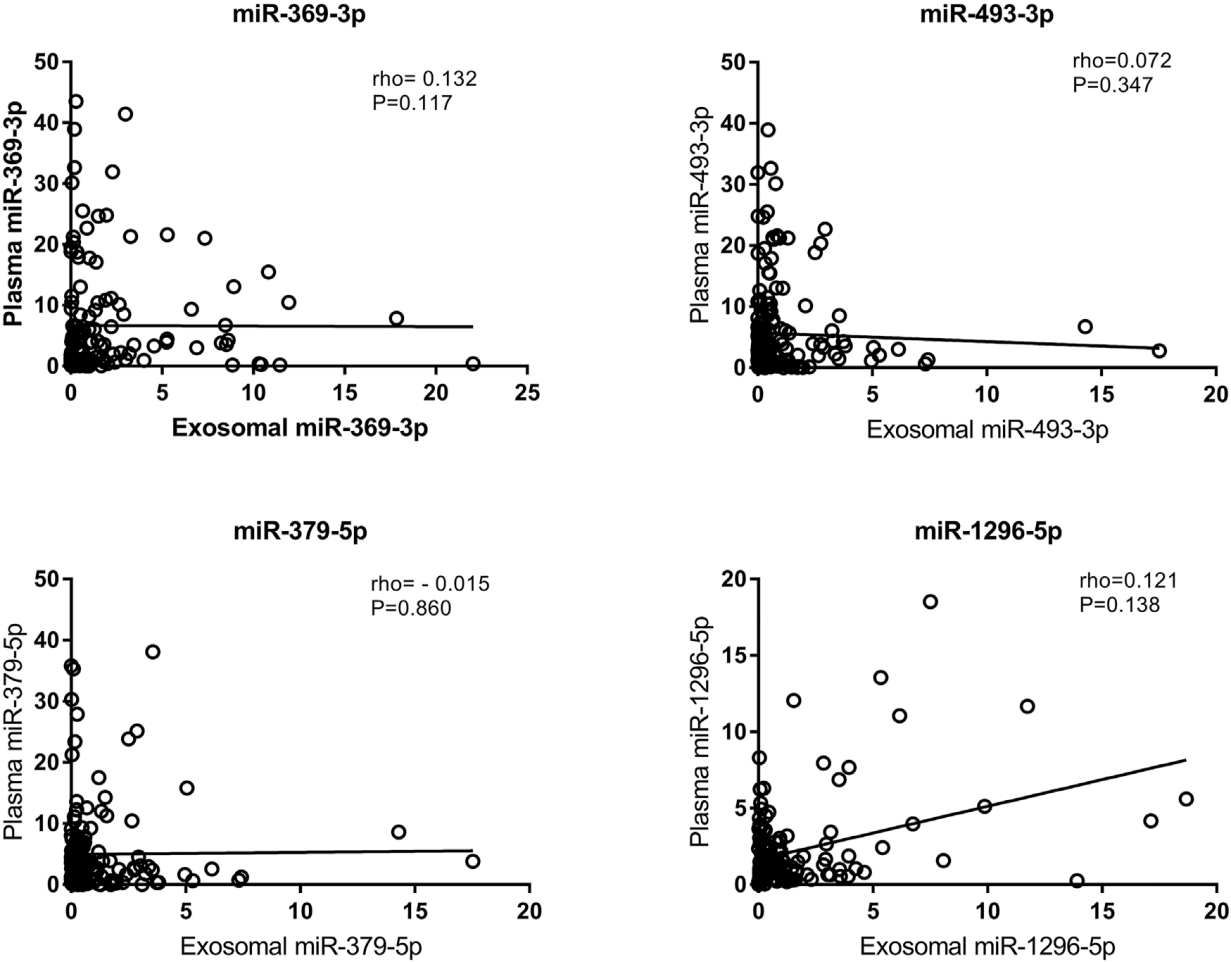
Correlation of exosomal miRNA expression levels with plasma miRNA expression levels as assessed using Spearman’s correlation coefficient (rho).

**FIGURE 6:**
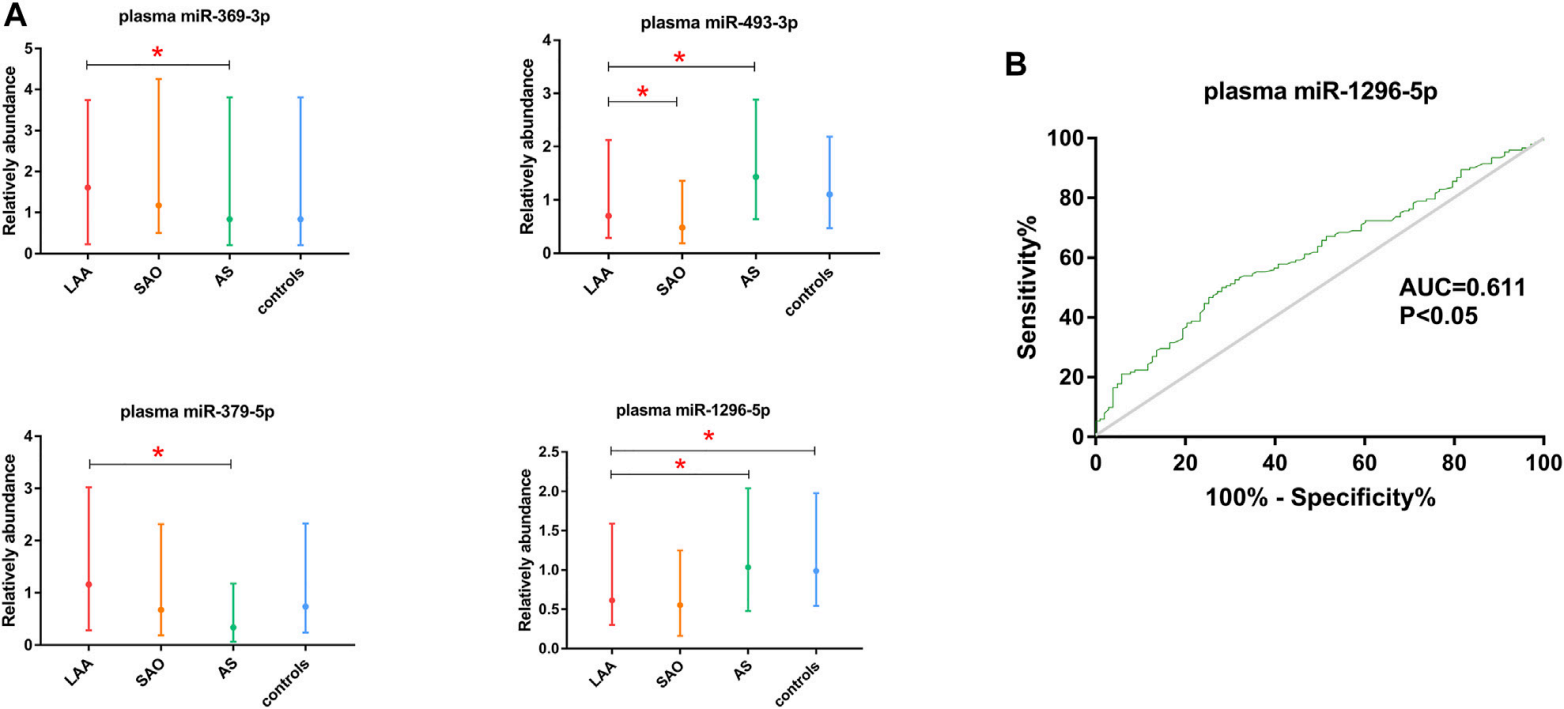
Validation of plasma miR-369-3p, miR-493-3p, miR-379-5p, and miR-1296-5p. **(A)**. qRT-PCR of miRNAs in plasma. **(B)**. ROC curve of plasma miR-1296-5p. The AUC was calculated and is shown in green (*p < 0.05).

We also tested the expression of miRNAs in plasma via qRT-PCR. According to the correlation analysis, there was no correlation between the expression of exosomal miRNAs and the expression of their counterparts in plasma (*p* > 0.05) (Figure 5). Plasma miR-369-3p, miR-493-3p, and miR-379-5p failed to distinguish between the LAA group and the control group (Figure 6A). In addition, miR-1296-5p in plasma exhibited an AUC of 0.611 (95% CI, 0.542–0.680), while the exosomal miRNA exhibited an AUC of 0.838 (NRI = 0.36) (Figure 6B). Thus, the diagnostic efficiency of miRNAs in exosomes is better than that of miRNAs in plasma.

### Diagnostic Efficiency of Composite Biomarkers

**FIGURE 7:**
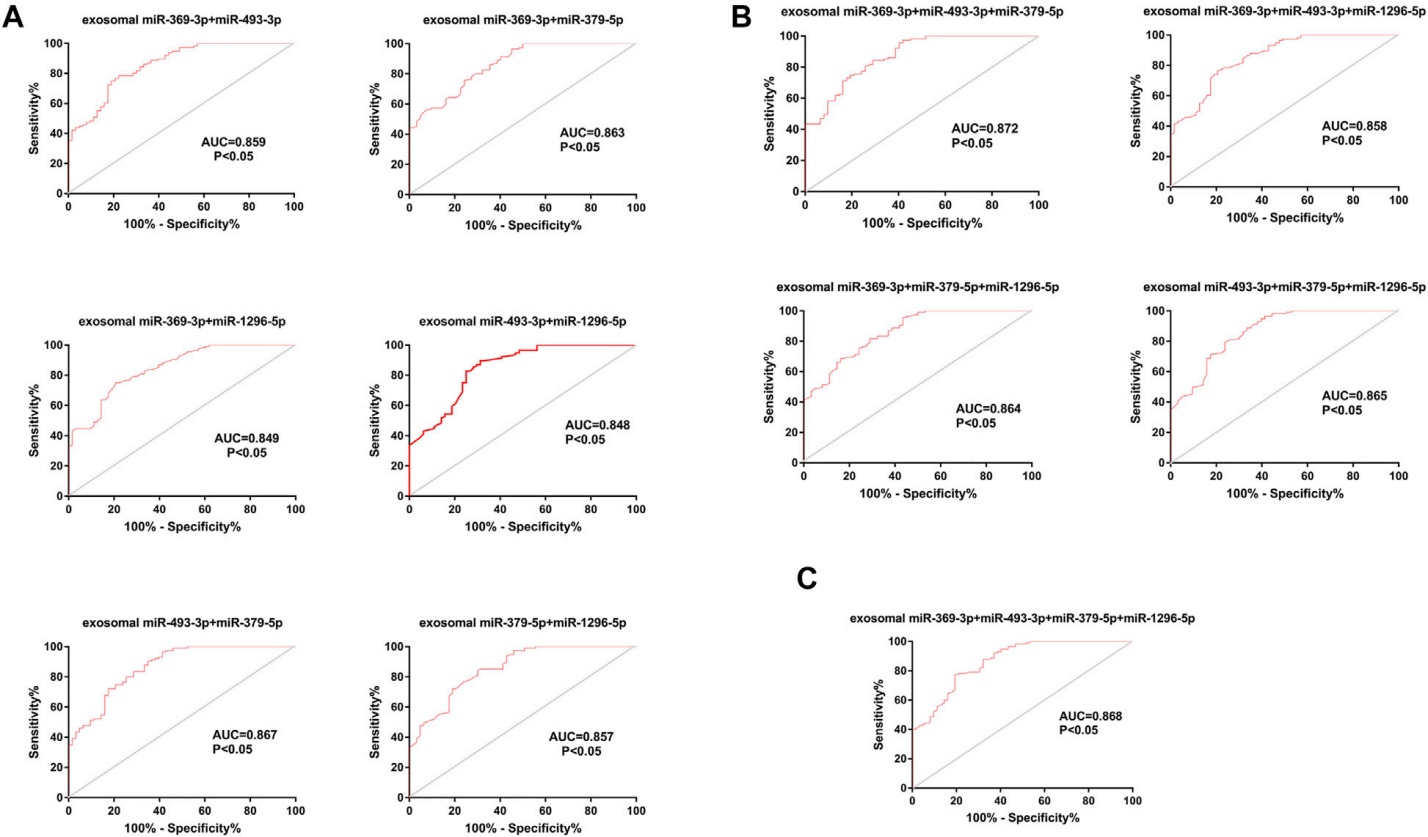
ROC curve analysis of composite biomarkers. **(A)**. Different combinations of two miRNAs. **(B)**. Different combinations of three miRNAs. **(C)** A combination of four miRNAs.

Considering that a single marker is not typically used for the final diagnosis in clinical practice, we tested the diagnostic efficiency of composite biomarkers by using a logistic model and ROC curve analysis (Min et al., 2019). The AUCs of exosomal miRNAs were as high as 0.867 (95% CI, 0.812–0.922) when different combinations of two biomarkers were used, higher than the value achieved for a single miRNA. Further, a combination of three miRNAs achieved an AUC of 0.872 (95% CI, 0.819–0.925); thus, adding miRNAs improved the diagnostic efficiency of a single biomarker to some extent. However, the AUC did not increase further a fourth miRNA was added (0.868 (95% CI, 0.813–0.922)) (Figures 7A–C).

### Correlations Between miRNA Expression Levels and NIHSS Scores

**FIGURE 8:**
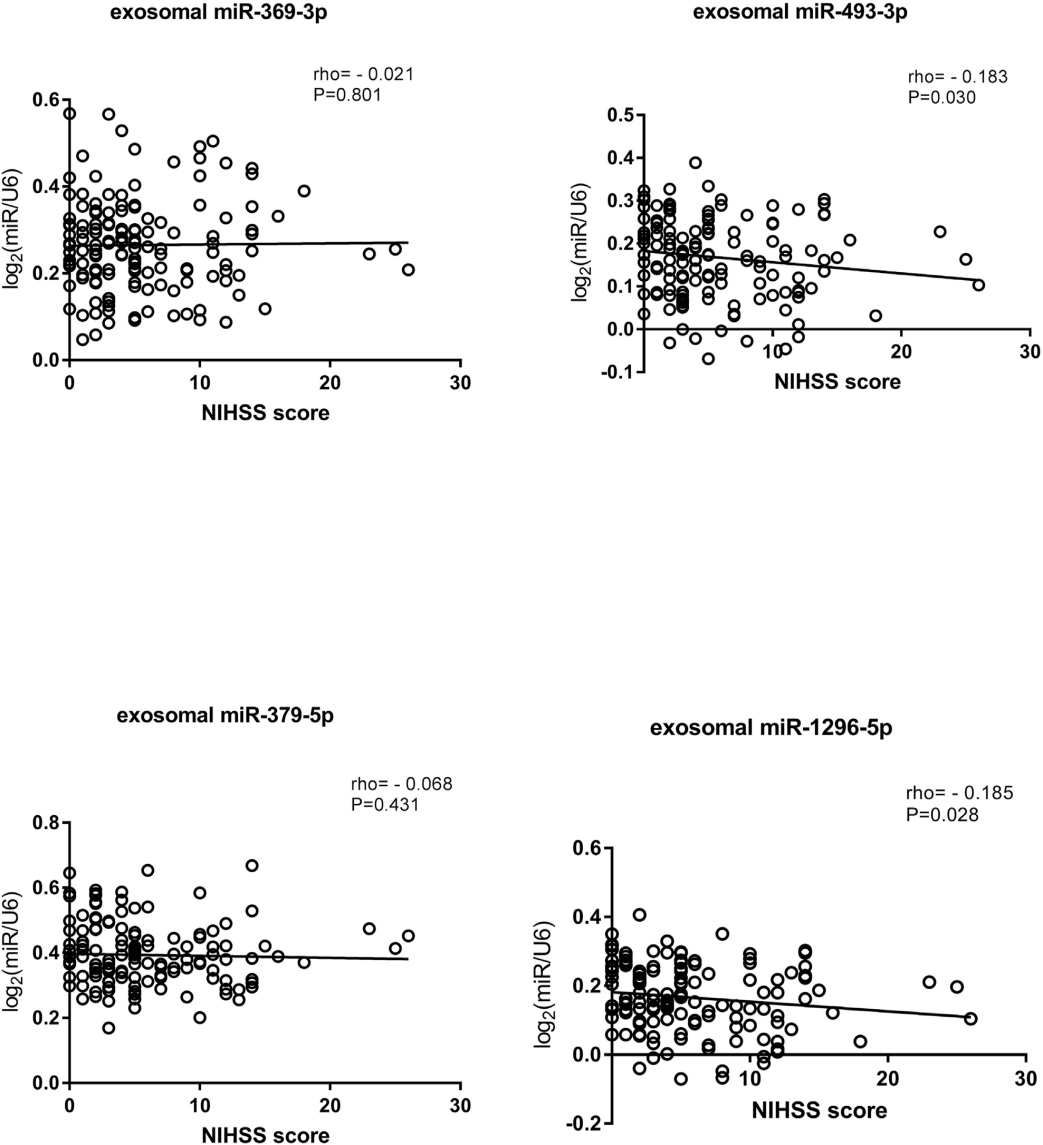
Correlations of exosomal miRNA expression levels with NIHSS scores as determined using Spearman’s correlation coefficient (rho).

In addition, we analyzed the relationship between NIHSS score and miRNA expression in exosomes. Patients’ conditions were clinically evaluated according to their NIHSS scores: a higher score indicated a more serious condition (Kwah and Diong, 2014). We classified patients according to their NIHSS scores as having mild stroke (NIHSS≤5) or moderate to severe stroke (NIHSS score>5) (Kvistad et al., 2019; Johnston et al., 2020). Spearman rank correlation was used to analyze the relationships between NIHSS scores and miRNA expression levels (Matsuura et al., 2016). The results suggested that the expression levels of exosomal miR-493-3p and miR-1296-5p were negatively correlated with the NIHSS score. Interestingly, exosomal miR-493-3p and miR-1296-5p expression was lower in patients with moderate to severe stroke than in patients with mild stroke (*p* < 0.05) (Figure 8).

## Discussion

In this study, we compared the diagnostic efficiency of miRNAs in exosomes and plasma among LAA, SAO, AS, and control groups. To the best of our knowledge, this is the first study to show that exosomal miRNAs can be used as biomarkers with better efficiency than plasma miRNAs for ischemic stroke. We screened out four identified exosomal miRNAs, namely, miR-369-3p, miR-493-3p, miR-379-5p, and miR-1296-5p, which are promising biomarkers for the diagnosis of LAA stroke, and their diagnostic efficiency is superior to that of their counterparts in plasma. In addition, we found that the expression levels of exosomal miR-493-3p and miR-1296-5p expression were negatively correlated with NIHSS score.

Ischemic stroke is a very urgent, serious, and heterogeneous disease, especially LAA stroke, which accounts for a considerable proportion of the incidence of ischemic stroke (Ornello et al., 2018). Therefore, proper identification and diagnosis of LAA are of vital importance for the success of follow-up treatment and for the quality of life of patients (Prabhakaran et al., 2015). The etiological diagnosis of ischemic stroke is mainly based on medical history, clinical evaluation, and cerebral angiography etc. (Jauch et al., 2017). When patients are unable to cooperate with examinations, diagnostic biomarkers can help solve the problem. Here, diagnosis of LAA stroke is undoubtedly time saving and cost saving, which is of great significance for the rational allocation of medical resources and further treatment (Tu et al., 2017b). In addition, we report a noninvasive method that can potentially be used for diagnosis. This method is feasible even when clinical resources are limited.

Exosomes are involved in cell-to-cell communication, material transport, immune responses, and other processes, and miRNAs are involved in multiple processes associated with stroke occurrence and progression (Krupinski and Slevin, 2013; Mirzaei et al., 2018; Raju et al., 2020). miRNAs are selectively packaged into exosomes, suggesting that they may carry specific information, and the numbers of exosomes and miRNAs secreted are affected by disease state and progression (Liu et al., 2019). Prior studies have shown that about 70% of miRNAs are expressed in the central nervous system, and a growing number of exosomal miRNAs play an important role in central nervous system diseases, such as ischemic stroke (Yu et al., 2021).

Previous studies have shown that miRNAs can serve as biomarkers for diagnosis of diseases, especially cancer (Min et al., 2019). In this work, we used high-throughput sequencing to gain an overall understanding of miRNA profiles. LAA is closely associated with inflammation and involves a cascade of inflammatory cytokines (Wolf and Ley, 2019). GO and KEGG analyses were used to screen out 5 miRNAs (miR-369-3p, miR-493-3p, miR-379-5p, miR-1296-5p, miR-1277-5p) according to the functions of their targeting genes. Among them, miR-369-3p is associated with low-density lipoprotein and monocyte-to-macrophage differentiation. And it has been reported to play a role in the inflammatory process, and its targeted genes are associated with inflammatory cytokines (Galleggiante et al., 2019). miR-1296-5p is associated with CAMs, white blood cell migration across endothelial cells, which are known as playing crucial roles in atherosclerosis (Libby et al., 2019). LAA is associated with a chronic inflammatory process involving CAMs (Zeng et al., 2021). Inflammatory cells can bind to cell adhesion molecules expressed by endothelial cells. In addition, during inflammation, cells migrate via chemotaxis in response to inflammatory cytokines (del Zoppo et al., 2000; Libby et al., 2019; Wolf and Ley, 2019). In addition, miR-493-3p has been found to modulate angiogenesis in a rat model of ischemic stroke (Li et al., 2016). miR-379-5p and miR-493-3p are related to metabolic pathways and fatty acid metabolism, which play essential roles in the pathophysiology of LAA (Libby et al., 2019). Besides, miR-493-3p is also related to antigen processing and presentation. Previous studies have shown that atherosclerotic plaque rupture correlates with the level of immune cells, and that antigen processing and presentation play important roles in regulating the function and level of immune cells (Kobiyama and Ley, 2018). In previous studies, miR-379-5p has been reported to be associated with autoimmune diseases of the central nervous system, multiple sclerosis, and its target genes are associated with cell death and inflammation, and miR-1296-5p inhibits liver cancer metastasis through the PI3K/Akt pathway and is downregulated in breast cancer (Xu et al., 2017; Baulina et al., 2018). What’s more, PI3K/Akt pathway has been confirmed to be associated with ischemic stroke (Qi et al., 2021). miR-1277-5p has been associated with inflammatory response and oxidative stress in previous study (Zhou et al., 2021). We speculate that the factors we screened play roles in the occurrence and progression of LAA. In total, 4 miRNAs (miR-369-3p, miR-493-3p, miR-379-5p, and miR-1296-5p) were selected for a large-sample validation phase. These factors have not often been studied in the context of cerebrovascular diseases, including ischemic stroke. Although these miRNAs have been identified as biomarkers for diagnosis and prognosis of other diseases, we are the first to identify them as biomarkers for ischemic stroke.

We analyzed our experimental results by calculating the relative expression levels of miRNAs based on the study of Rana Raoof et al (Raoof et al. (2018)). Our analysis revealed that the intergroup differences for exosomal miR-369-3p, miR-493-3p, miR-379-5p, and miR-1296-5p were significant. These miRNAs could distinguish the LAA group from the control group, the LAA group from the SAO group, and the LAA group from the AS group. The significant difference between the LAA and the control group distinguished patients with ischemic stroke from those without stroke. Differential expressions were also observed in LAA and SAO groups. However, the presence or absence of vascular stenosis was the main difference between the LAA and SAO groups, and SAO usually shows no signs of cortical dysfunction (Adams et al., 1993). Differentially expressed miRNAs between these two groups can be helpful for the classification of ischemic stroke. Additionally, LAA is generally caused by rupture of atherosclerotic plaques, and our results showed a significant difference between the LAA group and the AS group, which consisted of patients with unruptured atherosclerotic plaques, suggesting that plaques rupture may play a role in the pathogenesis of stroke (Chistiakov et al., 2015). Identification of the etiology of acute ischemic stroke (AIS) is of much importance for guiding the secondary prevention. That is, candidate biomarkers are important for early prevention, diagnosis and treatment of the disease. The screened candidate diagnostic biomarkers can potentially identify the etiology, in other words, the occurrence of stroke events caused by ruptured atherosclerotic plaque, which is helpful for early treatment of the etiology and guidance of clinical medication.

Notably, the experimental grouping in this experiment was more detailed than those in previous studies. Here, we included ischemic stroke patients, patients with AS, and healthy controls. Previous studies on ischemic stroke have divided participants into two groups: a stroke group and a control group (Jickling et al., 2014; Tiedt et al., 2017). We further refined the groups in this study. Since there are multiple subtypes of ischemic stroke, we focused on LAA and SAO. Moreover, strict exclusion criteria were adopted in the current study to ensure the rigor and accuracy of the experiment (Tiedt et al., 2017; Jia et al., 2019; Min et al., 2019). It has been previously reported that miRNAs in exosomes can be used as biomarkers of atherosclerosis in cardiovascular diseases (Liu et al., 2019). We made a more detailed grouping and applied exosomal miRNAs to the diagnosis of LAA stroke. As a result, we identified candidate biomarkers for LAA stroke. It is indicated that different exosomal miRNAs could be used as diagnostic biomarkers and play roles in different atherosclerotic diseases, while needs to be further studied. In this study, we focus on the diagnostic value of exosomal miRNAs in the acute stage of ischemic stroke. The occurrence of an ischemic stroke usually indicates the need for immediate evaluation of therapeutic interventions. Biomarkers, especially in the acute phase, can play a valuable role in the treatment of stroke. Longfei Jia, Steffen Tiedt, Li Min et al. have conducted cross-sectional studies in their respective fields, and similarly, we have adopted the same research methods to obtain alternative diagnostic biomarkers.

In order to validate the results, we calculated the AUCs of the four factors. The results suggested that compared with plasma miRNAs, the exosomal miRNAs (miR-369-3p, miR-493-3p, miR-379-5p, and miR-1296-5p) showed greater diagnostic efficiency with much higher AUCs. The AUC was also calculated for a panel of four potential biomarkers. A large number of studies have shown that the diagnostic efficiency of combined biomarkers is better than that of single biomarkers (Jia et al., 2019; Min et al., 2019). In line with these findings, composite biomarkers achieved higher AUCs than single biomarkers in this study. We verified the diagnostic efficiency of the four miRNAs in exosomes with a relatively large sample size of subjects to obtain clinical support. A study by Li Min et al. has verified that exosomal miRNAs are more effective than plasma miRNAs for the diagnosis of colon cancer (Min et al., 2019). Consistent with this finding, our experimental results showed that exosomal miRNAs were superior to their plasma counterparts for the diagnosis of LAA. In addition, there were significant differences in exosomal miRNAs among the four groups. In contrast, plasma miRNAs did not exhibit the same differential expression patterns, confirming that exosomes protect miRNAs from RNA-degrading enzymes in plasma. And according to our results, plasma miRNAs were not correlated with exosomal miRNAs. Diehl et al. found that miRNAs profiles in microvesicles were significantly different from those in maternal cells, suggesting that the selective packaging of miRNAs from cells into microvesicles is an active mechanism, which may indirectly prove the protective effect of exosomes, and partly explains the incorrelation between plasma and exosomal miRNAs in our results (Matsuura et al., 2016).

Prior studies on biomarkers for ischemic stroke have focused on proteins in plasma such as neuron-specific enolase and interleukin (AUC = 0.82 and 0.69 respectively) (Tiedt et al., 2017). According to Wang et al.’s study, exo-miRNAs perform better than traditional protein biomarkers, such as CEA and Cyfra21-1, in the diagnosis of non-small cell lung cancer (Wang et al., 2020). By contrast, in our study, we focused on exosomal miRNAs that can be used as diagnostic biomarkers in LAA stroke. Compared with previous studies, we investigated new factors for diagnostic biomarkers of LAA stroke that had not been previously studied. It is indicated in previous literature that miRNAs in plasma are easily degraded by RNase (Moreno-Moya et al., 2014). As a result, we not only studied the potential value of new factors in the diagnosis of ischemic stroke but also compared the diagnostic power of plasma factors and exosomal factors. The diagnostic efficiency of miRNAs in plasma vs. exosomes has been reported before (Min et al., 2019). Our results indicated that the diagnostic efficiency of exosomal miRNAs was superior to that of plasma miRNAs for ischemic stroke. We confirmed the protective effects of exosomes on miRNAs and the diagnostic value of exosomal miRNAs for ischemic stroke. Furthermore, we analyzed the identified miRNAs for the first time.

We also analyzed the correlations between miRNA expression levels in exosomes and NIHSS scores according to the methods in a previous study by Kentaro M. et al. (Matsuura et al., 2016). Early in the disease, a higher NIHSS score usually predicts a poorer prognosis to some extent (Powers, 2020). We classified ischemic stroke patients according to their NIHSS scores and found that exosomal miR-493-3p and miR-1296-5p levels were negatively correlated with NIHSS score. Therefore, we speculate that the expression levels of miRNAs in exosomes reflect the severity of disease to some extent. Although the two correlations were weak, the results provide new ideas for further exploration of the expression of miRNAs in the future. miRNA expression decreased with increasing disease severity, which confirms that miRNAs play important roles in disease progression. Our findings provide insights for determination of disease severity through quantitative detection of miRNAs in the future. Besides, it has been previously reported that baseline NIHSS score is an important parameter to predict the prognosis of acute ischemic stroke (Tu et al., 2017a; Tu et al., 2017b). Therefore, we speculated that exosomal miRNAs as biomarkers can be used for the prognosis analysis of LAA stroke, which may be helpful to improve the quality of life of stroke patients in the future.

There were some limitations to our study. First, we only included Chinese Han ethnicity as the research object of our study, which restricted the generalization of our experimental conclusions to other populations. In addition, more clinical trials are needed to confirm the diagnostic efficiency of the candidate miRNAs.

## Conclusion

In summary, our study sheds new light on the use of exosomal miRNAs as noninvasive diagnostic biomarkers for LAA. We also provide a new alternative diagnostic method. Exosomal miR-369-3p, miR-493-3p, miR-379-5p, and miR-1296-5p are potential biomarkers, and composite biomarkers achieve higher diagnostic efficiency than the single biomarkers, making them more suitable for clinical diagnosis. In addition, we found that the NIHSS score is negatively correlated with exosomal miRNA expression, which provides a new perspective for future studies (Xu et al., 2019).

## Data Availability

The data that support the findings of this study are available from the corresponding author upon reasonable request.

## Abbreviations

AS, atherosclerosis; AUC, area under the curve; CTA, computed tomography angiography; CAMs, cell adhesion molecules; CT, computed tomography; FC, fold change; GO, Gene Ontology; KEGG, Kyoto Encyclopedia of Genes and Genomes; FDR, false discovery rate; LAA, large-artery atherosclerosis stroke; MRI, magnetic resonance imaging; MRA, magnetic resonance angiography; NPV, negative predictive value; NIHSS score, National Institutes of Health Stroke Scale score; NTA, nanoparticle tracking analysis; PPV, positive predictive value; PBS, phosphate-buffered saline; qRT-PCR, quantitative real-time polymerase chain reaction; ROC, receiver operating characteristic curves; SAO, small artery occlusion stroke; SD, standard deviation; TOAST, Trial of Org 10,172 in Acute Stroke Treatment; TEM, transmission electron microscopy; TPM, transcripts per million.
