# Supplementary material for: Circulating Exosomal miRNAs as Novel Biomarkers Perform Superior Diagnostic Efficiency Compared With Plasma miRNAs for Large-Artery Atherosclerosis Stroke
Source: Front Pharmacol. 2021 Nov 26;12:791644. doi: 10.3389/fphar.2021.791644 (PMC8661454; doi:10.3389/fphar.2021.791644)
Supplement: Supplementary file 1 [file DataSheet1.PDF]

Approval document of medical ethics committee of the affiliated hospital of Qingdao university

Project Name: Associations of circulating exosomal non-coding RNAs with ischemic stroke in the Chinese Han Population

Assume the unit: Affiliated hospital of Qingdao university

Project leader: Xudong Pan

Project introduction: Ischemic stroke is considered to be a complex disease, which consists of a group of heterogeneous disorders with multiple genetic and environmental risk factors. Non-coding RNAs (miRNAs, circular RNAs and long non-coding RNAs) participated in various physiopathological processes. Recently, it is reported that exosomal non-coding RNAs are associated with some diseases, and these non-coding RNAs play a role in the diagnosis and treatment of diseases. However, diagnostic biomarkers of exosomal non-coding RNAs for ischemic stroke have rarely been studied. Therefore, the aim of this study was to investigate the association of circulating exosomal non-coding RNAs with ischemic stroke in the Chinese Han population.

Opinions of the medical ethics committee:

In this program, the rights and interests of the subjects are fully protected and meet the requirements of the medical ethics committee. Agree to the study plan.

Medical ethics committee of the affiliated hospital of Qingdao university (seal)

June 6, 2017
